# Important considerations when studying the impact of physical education on health in youth

**DOI:** 10.1186/1471-2431-14-75

**Published:** 2014-04-15

**Authors:** Laura Cañadas, Oscar L Veiga, David Martinez-Gomez

**Affiliations:** 1 Department of Physical Education, Sports and Human Movement, Faculty of Teacher Training and Education, Autonomous University of Madrid, Madrid, Spain; 2 Departamento de Educación Física, Deporte y Motricidad Humana, Facultad de Formación de Profesorado y Educación, Universidad Autónoma de Madrid, Campus de Canto Blanco, Ctra. de Colmenar Km 11, E-28049 Madrid, Spain

**Keywords:** Physical education, Health, Physical activity, Youth

## Abstract

Klakk et al. conducted an intervention study by increasing the frequency of physical education lessons in children aged 8 to 13 years, and they examined its effect on body fat during two school years. Physical education has potential to provide health in childhood and adolescence. For achieving these benefits, one of the most relevant aspects that need to be addressed during physical education classes is to provide students with high levels of physical activity. A well-recognized recommendation suggests that students should engage in moderate to vigorous physical activity for at least 50% of the time they spend in physical education classes. Therefore, it would be crucial to know what is happening during physical education classes before increasing their frequency. On the other hand, it seems that the main concern of health-related researchers is provide evidence on the impact of physical education on physical health outcomes (e.g. obesity), whereas other dimensions of health such as social, emotional, intellectual, and spiritual health are understudied. New evidence on the role of physical education on other health outcomes beyond physical health would also be important for the recognition of this curricular subject.

## Commentary

We have read with interest the recent study by Klakk and colleagues entitled “Effect of four additional physical education lessons on body composition in children aged 8–13 years – a prospective school study during two school years” [1]. Their main findings indicate that four additional lessons of physical education slightly decreased the prevalence of overweight and obesity in youth. Also, they found that the intervention had a greater effect in decreasing body fat among overweight and obese children at baseline. The study by Klakk et al. has important strengths (e.g. sample size, long-term intervention, imaging technics for measuring body fat), but some issues must be taken into consideration when interpreting its results as well as for designing future studies aimed to examine the impact of physical education on health.

As described by the authors, the single intervention was to increase the number of physical education lessons. In this study, the authors are based on the premise that a substantial increase in the frequency of physical education classes is a good opportunity to accumulate more physical activity at recommend levels (i.e. 60 minutes per day at moderate to vigorous intensity) and in turn, it will have benefits on health. However, we cannot assume that all physical educators have similar qualifications and skills to provide students with high levels of physical activity during their lessons. The main indicator of physical education quality in health-related physical education is guaranteeing that students are physically active in terms of duration and intensity in each lesson. The operational goal for this rationale would be to ensure at least 50% of physical education class time is spent in moderate-to-vigorous [2,3]. Importantly, the ability of physical educators of increasing physical activity at vigorous intensity would be essential for preventive purposes not only for obesity prevention but also for other chronic diseases [4]. Consequently, information on these characteristics of physical education in both control and intervention schools at baseline and during the follow-up would have been crucial.

On the other hand, physical education has potential to provide health in young people thorough (i) preparing students physical activity with the appropriate knowledge, skills, behaviors, and confidence to be physically active for life, and (ii) providing students with physical activity during physical education lessons, as commented above. However, being in good health is more than good physical health. The dimensions of health include not only physical health but also social, emotional, intellectual, and spiritual health [5]. For example, a systematic review by the Centers of Disease Control and Prevention [6] and recent studies indicate that physical education is associated with academic benefits such as improved memory, concentration, cognitive skills and school attitudes [7,8]. Klakk et al. examined the impact of a substantial change in physical education frequency on obesity. Though the results were weak but significant in decreasing the prevalence of overweight/obesity, it would have been important to know the impact of this intervention in physical education on other dimensions of health, beyond physical health.

## Response

By Heidi Klakk, Niels Wedderkopp and Lars Bo Andersen.

Thank you for the interest in our study – The CHAMPS study-DK.

The CHAMPS study-DK was an evaluation of a natural experiment, where a Danish municipality decided to establish sports schools with a tripling of physical education (PE) lessons, corresponding to six PE lessons per week. Given the nature of a natural experiment, the researchers had no influence or control of the content and intensity of the PE lessons besides the anticipation, that the teachers followed the age-related concept as taught to them in workshops during the first school year. The intention of introducing that concept was to enhance children’s joy of moving and their physical health, by improving their motor performance and fitness. Other researchers in the group currently prepare for publications on the effect on fitness, activity level and motor performance.

As put forward by Cañadas et al. it could be questioned whether substantial increase in the frequency of PE lessons without knowing/demanding an increase in intensity level would have the potential to actually lead to health benefits.

Preliminary analysis of physical activity (PA) levels (assessed with the GT3X Actigraph accelerometer) in schools and between schools in the CHAMPS study-DK show that PE lessons is the domain with the highest activity levels during the child’s school day. The intensity of PA in the PE lessons did not differ significantly between school types in our study, but tripling the duration of that domain – in a mandatory way – proved to be enough to have an impact on the children’s body composition expressed as prevalence and incidence of being overweight [1] and furthermore the level of cardio-vascular risk factors [9].

At neither intervention nor control schools the proportion of time spent in moderate-to-vigorous physical activity (MVPA) in PE lessons did not, with the chosen intensity and age related thresholds, exceed 50% as Cañadas et al. propose as an operational goal for PE lessons based on data from the CATCH study [3].

The observed health affects in the CHAMPS study-DK might to some extend be explained by a metabolic fitness effect despite intensity level and/or that the suggested operational goal (>50% of the time spent in MVPA) is not valid. In the CATCH study from 1996, PA levels were self-reported and/or observed. In a review on measurement issues, Ekelund et al. [10] conclude that there is low-to-moderate correlation (r =0.3-0.4) between self-reported and objectively measured PA levels and that intensity and duration might be overestimated by 72%. Furthermore Ekelund et al. stated that even with a more precise and objective measure of PA levels such as accelerometers, the proportion of children meeting a certain criteria (ie accumulation of >60 min of MVPA per day, or more than 50% of the time in PE spent in MVPA) vary considerably (from 1% to 100%). This variation is largely explained by the use of different intensity thresholds when defining MVPA [10]. Consequently it seems, that defining sufficient proportions and intensity levels of PE lessons is still a scientific challenge, even when PA levels are objectively measured.

Our findings are supported by studies on health benefits of cycling as it has been shown that high frequency (twice a day) with even shorter bouts (10–15 minutes) of MVPA can have beneficial health effects in children with or without changing their cardiorespiratory fitness [11,12].

In summary we therefore still put forward that, in the CHAMPS study-DK, a substantial increase in the frequency of PE lessons, regardless of knowing the intensity levels, did have a considerable and valuable public health effect in healthy children.

We do agree, that other aspects of children’s well-being are important as well, but was not the scope of this publication. The CHAMPS study-DK is an on-going cohort study (until now 6 years of follow up) and measurements of cognition, implementation and sustainment are planned for, but not yet completed.

## Competing interests

The authors declare that there is no competing interest regarding the material discussed in this commentary.

## Authors’ contributions

LC and DMG –prepared the first draft of manuscript. DMG and OV– revised the manuscript. LC –prepared bibliographical background. All authors read and approved the final manuscript.

## Pre-publication history

The pre-publication history for this paper can be accessed here:

http://www.biomedcentral.com/1471-2431/14/75/prepub
